# Structural and Functional Asymmetries in Male Basketball Players: A Cross-Sectional Analysis of Body Composition, Bone Status, and Performance

**DOI:** 10.3390/jfmk10030359

**Published:** 2025-09-18

**Authors:** Dimitrios Pantazis, Dimitrios Balampanos, Alexandra Avloniti, Theodoros Stampoulis, Maria Protopappa, Christos Kokkotis, Konstantinos Chatzichristos, Panagiotis Aggelakis, Maria Emmanouilidou, Nikolaos-Orestis Retzepis, Nikolaos Zaras, Dimitrios Draganidis, Ioannis G. Fatouros, Maria Michalopoulou, Antonis Kambas, Athanasios Chatzinikolaou

**Affiliations:** 1 Department of Physical Education and Sport Science, School of Physical Education, Sport Science and Occupational Therapy, Democritus University of Thrace, 69100 Komotini, Greece; dpantazi@phyed.duth.gr (D.P.); dimibala10@phyed.duth.gr (D.B.); alavloni@phyed.duth.gr (A.A.); tstampou@phyed.duth.gr (T.S.); mprotopa@phyed.duth.gr (M.P.); ckokkoti@affil.duth.gr (C.K.); kchatzic@phyed.duth.gr (K.C.); pangelak@phyed.duth.gr (P.A.); maemmano@phyed.duth.gr (M.E.); nretzepi@phyed.duth.gr (N.-O.R.); nzaras@phyed.duth.gr (N.Z.); michal@phyed.duth.gr (M.M.); akampas@phyed.duth.gr (A.K.); 2 Department of Physical Education and Sport Science, School of Physical Education, Sport Science and Dietetics, University of Thessaly, 43100 Trikala, Greece; ddraganidis@uth.gr (D.D.); fatouros@uth.gr (I.G.F.)

**Keywords:** asymmetries, vertical jump, basketball, injury risk

## Abstract

**Background:** Basketball is a high-intensity, multidirectional sport involving frequent jumping, sprinting, and rapid changes of direction, which may expose the musculoskeletal system to varying and potentially asymmetric mechanical demands. The mechanical loading associated with basketball-specific movements may also serve as a consistent osteogenic stimulus, potentially leading to side-specific adaptations in body composition and bone characteristics. Long-term participation in basketball may lead to functional and structural asymmetries between the lower and upper limbs, potentially increasing the risk of injury and impacting performance. This study aimed to investigate structural and functional asymmetries in male basketball players using body composition, health, and performance-related measures. **Methods:** Thirty-eight right-handed basketball players (age: 21.1 ± 2.8 years; body mass: 86.2 ± 9.2 kg; height: 1.91 ± 8.3 cm) were assessed in a single testing session. The evaluation included bioelectrical impedance analysis (BIA), dual-energy X-ray absorptiometry (DXA), single-leg countermovement rebound jumps (CMRJs), and handgrip strength testing. **Results:** Significant interlimb differences were observed in lean mass and the phase angle for both the arms and legs. Performance differences favored the left leg in terms of maximum jump height (12.0 ± 17.5%, *p* = 0.001) and reactive strength index (RSI), whereas the right arm exhibited greater grip strength than the left (6.4 ± 5.9%, *p* = 0.001). DXA analysis revealed significant asymmetries in bone parameters, including bone mineral density (BMD) of the trochanter (1.81 ± 5.51%, *p* = 0.031, dz = 0.37), total hip (1.41 ± 4.11%, *p* = 0.033, dz = 0.36), and total arms (–1.21 ± 2.71%, *p* = 0.010, dz = 0.43), as well as bone mineral content (BMC) in total arms (–2.16 ± 5.09%, *p* = 0.012) and total legs (1.71 ± 3.36%, *p* = 0.002, 0.54). **Conclusions:** These findings suggest that basketball may induce both functional and structural adaptations, likely due to repetitive unilateral loading and sport-specific movement patterns. However, individual variability and the use of diverse assessment methods may complicate the detection and interpretation of asymmetries. Coaches and practitioners should monitor and address such asymmetries to reduce injury risk and optimize performance.

## 1. Introduction

Basketball is an intermittent team sport characterized by frequent changes in activity patterns and intensity, combining high-intensity, multidirectional actions, such as sprinting, jumping, rapid acceleration, deceleration, and changes of direction (COD) or shuffling, interspersed with lower-intensity activities, such as jogging, walking, or standing, which are seamlessly incorporated into basketball-specific skills like shooting, passing, and dribbling [1,2]. During an official basketball match, elite players perform an average of 2.38 ± 0.63 decelerations/min, 2.19 ± 0.84 accelerations/min, 1.11 ± 0.53 jumps/min, and 10.62 ± 3.26 changes of direction (COD)/min, underlining the high-intensity physical demands of the sport and the potential cumulative stress on players’ musculoskeletal systems [1]. Due to the high impact and multidimensional loads imposed on the body, basketball is considered a high-impact osteogenic team sport [3,4]. Basketball players exhibit a higher bone mineral density (BMD) than non-athletes, as well as athletes engaged in low-impact sports, such as swimming, and other high-impact team sports, including soccer and volleyball [3,4]. This could likely be attributed to the higher rate of high-intensity running, sprinting, deceleration, and changes of direction, combined with the hardwood surfaces typically used in basketball, which may generate greater ground reaction forces than other surfaces [5]. These characteristics promote higher forces and repetitive loading on the skeletal system, offering an enhanced osteogenic stimulus [3]. Under a strain adaptation framework, bone and soft tissue remodel locally to limb-specific loading histories; thus, segmental BMD/BMC and body composition can index cumulative, side-specific mechanical exposure rather than whole-body status. These mechanical loading patterns are particularly impactful during the late childhood and post-pubertal years, when bone remodeling is highly responsive, underlining its importance in fracture prevention both during growth and later in life [6,7].

These high-intensity actions, often involving basketball-specific movements such as offensive and defensive cuts or jump landings, impose significant biomechanical load on the body, including sagittal plane bending and knee valgus, which predispose athletes to injury risk in the lower extremities [8]. The relationship between injury occurrence and inter-limb asymmetries has been extensively studied, with evidence suggesting that functional asymmetries exceeding 10–15% are associated with an increased risk of injury, impaired physical performance, and reduced efficiency in sport-specific tasks [9,10,11]. Consequently, assessing and monitoring inter-limb asymmetries has become increasingly popular among strength and conditioning coaches, athletic trainers, medical staff, and researchers. Specifically, increased lower-limb power asymmetries have been linked to reductions in jump height, sprint speed, and COD performance [9,12,13]. Furthermore, inter-limb asymmetry is widely used to quantify functional deficits through all stages of injury rehabilitation, leading to a safe return to play and preventing re-injury [14,15,16].

The prevalence of asymmetries has been extensively investigated in basketball players, with varying findings on the effects of their presence on athletic performance [17,18,19,20,21], body composition, bone health [7,12], and muscle activation patterns [22] both during basketball-specific movement patterns and training sessions/matches [23,24]. Positional differences have been observed, with forwards and centers exhibiting greater asymmetry than guards in single-leg jump performance and muscle activation during training, particularly in competition [17,22]. The level of players also appears to be a consideration, with higher-level players showing greater symmetry compared to those at a lower competitive level [17]. Research on elite youth athletes has demonstrated that asymmetry is multifactorial, suggesting the need for assessment across a range of physical abilities [18]. Furthermore, injury history may be a more critical determinant of asymmetry than the physical demands of the sport itself in professional basketball players [21]. Recently, the focus on asymmetries in basketball has expanded beyond the risk of injury to their potential impact on performance. Research findings have linked single-leg countermovement jump performance (SLCMJ) asymmetries to linear sprint and agility, as well as hip and trunk torque development rate (RTD) to agility [25]. In addition, Bell et al. [12] demonstrated that lean mass asymmetry significantly affects peak force and power asymmetries during jumping. Asymmetries in power exceeding 10% are associated with reductions in jump height, resulting in average performance deficits of 9 cm. However, low levels of asymmetry (generally below 15%) failed to reveal any correlation between unilateral jump performance in all three axes and multi-directional sprinting [26].

Research focusing on detecting asymmetries during fundamental movements, basketball practices, and games revealed differences in curvilinear movements, where the outside leg is loaded more than the inside leg. Interestingly, no asymmetries were found during accelerations, decelerations, jumps, or small-sided games [23]. Furthermore, a study of 12 professional male players from the Spanish first division suggested that basketball is a symmetrical sport, with no significant asymmetries found in different tasks, game situations, or player positions during training sessions and preseason matches [24]. Importantly, task-level symmetry during isolated sessions does not preclude the accumulation of cumulative structural asymmetry over seasons due to limb-specific exposure. Although the current study demonstrates that during training sessions or competition, basketball players perform symmetrical movements [24], asymmetries in physical abilities are still evident [17,18,19,21]. In this context, basketball imposes limb-specific loading through contralateral take-offs, curvilinear running, and unilateral landings; under strain-adaptation principles, such exposure is expected to leave segmental signatures in structure that whole-body metrics miss, with DXA-derived BMD/BMC indexing longer-term skeletal remodeling and BIA-derived lean mass reflecting medium-term soft tissue status. To the best of our knowledge, few studies have quantified segmental asymmetries in body composition and bone status in basketball, and integrated evaluation of both upper and lower limbs alongside unilateral performance within the same cohort remains scarce [7,12,27]. Because power-oriented tests detect asymmetries more sensitively than traditional strength measures [28], lower-limb performance was indexed with the single-leg countermovement rebound jump (SLCMRJ), whereas handgrip strength served as a standardized unilateral upper-limb index. Quantifying the magnitude and direction of structural asymmetries together with these task-relevant outcomes permits limb-level appraisal of the coupling between structure and function and supports task- and segment-specific interpretation of asymmetry thresholds [12,25,28]. Although this study is cross-sectional and does not evaluate injury outcomes, the resulting limb-resolved map provides a translational baseline for individualized monitoring and a foundation for prospective work. Therefore, this study aimed to investigate structural and functional asymmetries in the upper and lower limbs of basketball players.

Based on this rationale, we prespecified the following directional hypotheses: in right-handed players, (H1) the right arm would show greater BIA-derived lean mass, greater DXA-derived BMD/BMC, and higher handgrip strength than the left; (H2) the left leg would show greater DXA-derived BMD/BMC and superior single-leg countermovement rebound jump (SLCMRJ) performance (greater height and RSI, shorter contact time) than the right; and (H3) signed inter-limb structural asymmetry would co-vary with homologous functional asymmetry within limb pairs, assessed via correlations of signed percent differences (right−left).

## 2. Materials and Methods

### 2.1. Participants

Thirty-eight professional basketball players were recruited for this study. The descriptive characteristics, including anthropometric and performance-related variables, are presented in Table 1. To be eligible for the study, participants had to meet the following inclusion criteria: (1) right-handedness; (2) no history of musculoskeletal injuries, illnesses, or metabolic disorders affecting performance for the last 12 months; (3) no use of medications six months prior to the study; (4) non-smoking status; and (5) regular participation in at least 10 h of basketball practice, two full-body weightlifting sessions per week, and at least one competitive game per week in national leagues. Each participant received detailed information regarding the benefits, risks, and potential discomforts associated with the study and provided written informed consent before participation. For underage participants, consent was obtained from parents or legal guardians. All procedures complied with the 2024 Declaration of Helsinki, the eighth revision, approved at the 75th Meeting in Helsinki, and ethical approval was obtained from the Ethics Committee of the Department of Physical Education and Sport Science, Democritus University of Thrace (Protocol No: DUTH/EHDE/38799/317-31 January 2025).

### 2.2. Study Design

A cross-sectional study was conducted in January 2025 during a single week without official game obligations. All measurements were completed within that week, and all testing sessions were scheduled in the morning hours. Participants were national-level basketball athletes competing in Greece’s Third Division; playing positions were recorded (19 backcourt, 19 frontcourt). To ensure sufficient sport-specific exposure, an a priori inclusion criterion of at least 6 years of organized basketball training was applied. Each athlete completed two laboratory sessions. Session 1 consisted of anthropometric measurements (stature and body mass) followed by bioelectrical impedance analysis (BIA) and dual-energy X-ray absorptiometry (DXA), in that order, to assess body composition and bone health. Session 2 evaluated performance, assessing lower-limb strength and explosiveness via countermovement rebound jumps (CRMJ) performed as triple bilateral and double unilateral sequences, and upper-limb strength with the handgrip (HG) test. From the single-leg CRMJ (SLCMRJ), we derived jump height, reactive strength index (RSI), contact time (CT), and an average height metric across the prescribed sequence, as per protocol. A familiarization week preceded data collection, during which participants practiced CRMJ/SLCMRJ and HG daily to standardize technique. During testing, each outcome was obtained from three maximal trials, and the best trial was retained for analysis. The order of limb testing (right/dominant vs. left/non-dominant) and the sequence of trials/tests were randomized to minimize order and crossover effects. To contextualize competitive demands, external load during training sessions and matches was monitored throughout the season using inertial measurement units (IMUs). For the present manuscript, we report representative weekly values from pre-season, in-season, and the transition period descriptively (Table 2). These IMU data (averages from 18 players, each value reflecting the cumulative load of one typical training week per phase) were used solely for context and were not included in the primary asymmetry analyses.

### 2.3. Testing Procedures

#### 2.3.1. Anthropometric Assessments

Bioelectrical impedance analysis (Charder MA801, multifrequency, 8-point electrodes) was used to estimate total body water and body composition. The device records resistance (R) and reactance (Xc) and automatically reports phase angle (PA), computed internally as arctan(Xc/R) × 180/π. Whole-body and segmental outputs were obtained.

Height was measured using the SECA 700 Stadiometerer (SECA, Hamburg, Germany), with participants standing barefoot on the platform. The adjustable headpiece was positioned on the top of the participant’s head, and their height was recorded and rounded to the nearest millimeter. Before conducting the Bioelectrical Impedance Analysis (BIA), participants were instructed to remove any metal objects and empty their bladders to ensure maximum measurement accuracy. The analysis was performed using the Charder MA801 (Charder, Taichung, Taiwan), a multifrequency device equipped with 8-point standing electrodes. The device operated across a range of frequencies—5 kHz, 20 kHz, 50 kHz, 100 kHz, and 250 kHz—each selected to interact with different tissues, allowing for a precise breakdown of fat mass, lean mass, and tissue mass. Measurements were conducted both for the body as a whole and segmentally, providing detailed insights into specific regions such as the arms, legs, and trunk. Fat mass was assessed by measuring the body’s resistance to electrical current, as fat tissue exhibits higher resistance due to its low water content. Lean mass, comprising muscles and organs, was measured through the lower resistance of these water-rich tissues, allowing for an accurate assessment of the body’s non-fat, active components.

Participants were instructed to stand barefoot on the platform, holding the electrodes in an upright position. A low-intensity current was passed through their bodies, and the device measured resistance and reactance in order to estimate body composition. To reduce variability, measurements were taken by trained staff in a controlled setting. Total body weight was recorded using the same device, followed by a calculation of BMI using the standard formula: weight in kilograms divided by height in meters squared. The procedures were implemented in line with the updated positioning guidelines as detailed by Balampanos et al. [29].

#### 2.3.2. Bone Health Assessment

DXA was conducted using a GE Healthcare Lunar DPX NT Bone Densitometer (Madison, WI, USA) to evaluate bone health under standardized conditions [30]. Participants removed all metallic items to avoid scan interference. The total body and dual femur scans, lasting approximately 35 min, exposed participants to a minimal radiation dose (0.01–0.03 mSv), significantly lower than that of a standard chest X-ray (0.1 mSv). The system utilized dual-energy X-ray beams with a constant potential source (76 kVp) and a K-edge filter, ensuring dose efficiency and precise differentiation between bone and soft tissue. Pencil-beam technology utilizes a narrow X-ray beam and a single detector that moves in a raster pattern, thereby minimizing geometric distortions and measurement errors. Quality assurance was maintained through daily phantom calibration, while enCORE software version 14.10.022 (GE/Lunar) automatically selected the scan mode and performed region-of-interest (ROI) analysis to reduce operator variability.

For whole-body scanning, participants were positioned according to the NHANES protocol: their arms were placed straight or slightly angled with palms down, their hands were isolated from the torso, their legs were extended, their feet were in a neutral position, and their ankles were strapped. The head was positioned with the chin in a neutral position, facing upward [31]. For dual femur scanning, participants maintained their arms and legs straight and parallel, with toes pointing upwards. Lower limb alignment was carefully ensured. Internal rotation of approximately 15–20° was applied using a foot-positioning device secured with a strap to prevent motion. This alignment positioned the femoral neck parallel to the scan table, minimizing the visibility of the lesser trochanter, as recommended. Legs were adjusted to be parallel to the edge of the table, and care was taken to avoid soft tissue being mistakenly identified as bone in regions caudal to the femoral neck. If the femoral neck overlapped with the ischium, the ischium was excluded (neutralized) from the analysis. These procedures followed updated positioning guidelines as described by Slart et al. [31].

Regions of interest (ROIs) for the whole-body scan included the total arms and total legs. In the hip, ROIs included the femoral neck, upper femoral neck, lower femoral neck, Ward’s region, greater trochanter, femoral shaft, and total hip. ROI placement for both the femoral neck and total body regions was visually checked and adjusted as necessary. All scans were analyzed using enCORE software, which automatically identified ROIs for each body region. If the automated segmentation did not comply with standardized anatomical guidelines, corrections were manually performed by trained examiners. The following DXA-derived variables were extracted separately for each side (left and right): bone mineral content (BMC) and bone mineral density (BMD = BMC/area) for the total arm, total leg, femoral neck, upper neck, lower neck, Ward’s region, greater trochanter, femoral shaft, and total hip.

#### 2.3.3. Lower Limb Power and Upper Body Strength

Prior to the assessment protocol, subjects completed a standard 20 min warm-up, including 5 min of core activation, 5 min of light running, 5 min of dynamic stretches targeting both the lower and upper body, and 5 min of protocol simulation exercises. These exercises consisted of both bilateral and unilateral countermovement jumps to develop lower-body strength, as well as push-ups to develop upper-body strength. After the warm-up, participants performed three trials for each assessment on a contact mat (EzeJump Swift Performance, Brisbane, Australia), with the order of execution being randomized. Variables measured in the unilateral and bilateral countermovement rebound jumps (CMRJ) were jump height (JH), average JH, contact time (CT), average CT, and reactive strength index (RSI). JH was calculated using the following formula based on the duration of the flight phase (tf): h = 1/8 g t^2^f, where tf is the duration of the flight phase and g is the acceleration due to gravity (9.81 m/s^2^). CT was measured in milliseconds, recording the time the foot or feet were in contact with the mat between rebound jumps. RSI was calculated by dividing JH by CT. The analysis considered the trial with the highest average JH test performed by each participant.

##### Countermovement Rebound Jump Testing (Unilateral and Bilateral)

Subjects performed two variations of the countermovement rebound jump (CMRJ). For the unilateral CMRJ, participants started the test standing in a unilateral position on a contact mat, with the test leg fully extended and the alternate leg bent at 90 degrees at both the hip and knee, and their hands on their hips. They were instructed to jump as high as possible, landing on the same leg to ensure a low contact time and a quick second rebound jump. Hands remained on the hips, and swinging the opposite leg was prohibited. An experienced examiner supervised the trials, providing a minute’s rest between them and requesting retesting if the instructions were not followed [32,33,34]. For the bilateral CMRJ, subjects initiated the test by standing in a comfortable, bilateral position with legs fully extended and feet positioned hip-width apart over the center of the contact mat [35]. Their hands were placed on their hips. The execution technique was identical to the unilateral CMRJ, with the modification that participants were instructed to jump and land on both feet simultaneously. After the initial countermovement jump, they were required to perform two rebound jumps, aiming to achieve a high jump height and a low contact time.

##### Unilateral Hand Grip Strength Test

Handgrip strength was assessed using a calibrated hydraulic hand dynamometer (Charder MG4800, Taichung City, Taiwan). Each participant remained seated upright in a height-adjustable chair to ensure accuracy. An experienced examiner positioned each participant with supported legs, adducted and neutrally rotated shoulders, bent elbows at 90 degrees, and forearms and wrists in a neutral position, with allowable wrist extension between 0 and 30 degrees. The elbow flexion angle was measured using a goniometer, and the subject’s arm was positioned on a table to support the weight of the dynamometer. HG strength was assessed in both the dominant and non-dominant arm, with a 5 min rest period between tests in each arm. The assessment consisted of three maximum isometric contraction trials per hand, each lasting 5 s, with a 1 min rest period between tests. The highest value recorded from these trials was considered the maximum voluntary contraction (MVC) [36].

#### 2.3.4. External Load Monitoring

External-load monitoring was included to document the seasonal mechanical exposure of the cohort and to contextualize athletes’ training status during the assessment window. These metrics are presented descriptively only and were not analyzed against asymmetry outcomes; their role is to provide context for interpreting structural findings (e.g., bone health) and to reduce the likelihood that results reflect short-term detraining. Throughout the season, microsensor data were recorded via IMU microsensors and downloaded after each session to a system computer for analysis using Kinexon software (Kinexon Perform IMU 12.0, KINEXON Precision Technologies, Munich, Germany). The IMU microsensor included a 3-axis accelerometer with a range of ±16 G at 1 kHz (provided at 100 Hz), a 3-axis gyroscope with a range of ±4000 deg/s at 200 Hz, and a 3-axis magnetometer with a range of ±16 μT at 100 Hz. Sensor data were captured continuously during training sessions and exported for analysis using Kinexon Perform software (version 12.0). EL metrics included accumulated acceleration load (AAL; Player Load: within-device CV = 0.91–1.05%, between-device CV = 1.02–1.90%), calculated as the square root of the sum of the squares of the instantaneous rate of change of acceleration on each of the three vectors (X, Y, and Z axes) and divided by 100 [37,38]. The total distance covered was the estimated distance the athletes travelled throughout the session. Mechanical load (ML) was derived by accumulating all the instantaneous acceleration and deceleration samples in the X and Y planes. Jump load (JL) was calculated using the equation JL = M × g × vertical displacement, where M is the mass (kg), g is the gravitational constant (m/s^2^), and vertical displacement is the jump height (meters). Jump events were also identified and quantified using Kinexon’s athlete tracking system, which integrates inertial sensor data as described by Pantazis et al. [39].

### 2.4. Statistical Analysis

All variables are reported as means and standard deviations (SDs). Interlimb asymmetries were quantified as a percentage difference between limbs, specifically using the Limb Symmetry Index (LSI), based on the best trial performances, following the formula proposed by Bishop et al. [40]. To facilitate this calculation, a modification using the “IF” function was implemented in Microsoft Excel. A positive LSI indicated that the right limb had a higher value, whereas a negative LSI indicated dominance of the left limb. Prior to performing parametric analyses, statistical assumptions were checked. Normality was assessed via skewness and kurtosis statistics, with Z-scores (skewness/kurtosis divided by their standard errors) within ±1.96 considered indicative of approximate normality, which was satisfied by the majority of variables. Homogeneity of variances was examined using Mauchly’s test of sphericity, and where the assumption was violated, Greenhouse–Geisser corrections were applied. Paired-sample t-tests were performed to assess side-to-side differences, comparing the left and right limbs separately for the upper (left vs. right arm) and lower extremities (left vs. right leg). Effect sizes (Cohen’s d) were reported alongside *p*-values to quantify the magnitude of observed differences. Correlations between variables were assessed using Pearson’s r, with corresponding effect sizes interpreted according to conventional thresholds. A one-way repeated measures ANOVA was used to assess differences in external load metrics (Accumulated Acceleration Load, Distance, Mechanical Load, Jump Load, and Jumps) across four phases of the season (Preseason, Early In-Season, Late In-Season, Offseason). Where significant main effects were found, Bonferroni post hoc tests were applied to examine pairwise comparisons. Partial eta squared (η^2^) was reported as a measure of effect size. All data were analyzed using IBM Corp. Released 2023. IBM SPSS Statistics for Windows, Version 29.0.2.0. Armonk, NY, USA: IBM Corp. Statistical significance was set at *p* ≤ 0.05.

## 3. Results

All participants successfully completed all measurements without any reported injuries. Table 3 presents a detailed analysis of body composition between limbs, derived from BIA measurements. Significant differences were identified between the upper limbs for phase angle (Left: 6.82 ± 0.67°, Right: 7.11 ± 0.62°, *p* = 0.000) and lean mass (Left: 4.43 ± 0.53 kg, Right: 4.54 ± 0.57 kg, *p* = 0.000). Likewise, significant differences were also observed between the lower limbs in phase angle (Left: 6.28 ± 0.67°, Right: 6.37 ± 0.68°, *p* = 0.040). Differences in lean mass of the legs (*p* = 0.080), fat mass in the arms (*p* = 0.422), and fat mass in the legs (*p* = 0.584) were not statistically significant.

Table 4 presents the bone mineral density (BMD) and bone mineral content (BMC) comparisons between limbs. Significant differences were observed in total arm bone mineral density (BMD) (Left: 1.015 ± 0.076 g/cm^2^, Right: 1.028 ± 0.081 g/cm^2^, *p* = 0.010), total arm bone mineral content (BMC) (Left: 265.19 ± 40.29 g, Right: 271.13 ± 41.49 g, *p* = 0.012), total leg BMC (Left: 816.23 ± 105.63 g, Right: 801.42 ± 106.88 g, *p* = 0.002), trochanteric BMD (Left: 1.216 ± 0.137 g/cm^2^, Right: 1.192 ± 0.138 g/cm^2^, *p* = 0.031), and total hip BMD (Left: 1.424 ± 0.154 g/cm^2^, Right: 1.402 ± 0.146 g/cm^2^, *p* = 0.033). No statistically significant differences were found for BMD in the total legs (*p* = 0.083), neck (*p* = 0.462), upper neck (*p* = 0.319), lower neck (*p* = 0.790), Ward’s region (*p* = 0.705), and shaft (*p* = 0.232) or for BMC in the neck (*p* = 0.304), upper neck (*p* = 0.225), lower neck (*p* = 0.496), Ward’s region (*p* = 0.493), trochanter (*p* = 0.279), shaft (*p* = 0.417), and total hip (*p* = 0.438).

**Figure 1:**
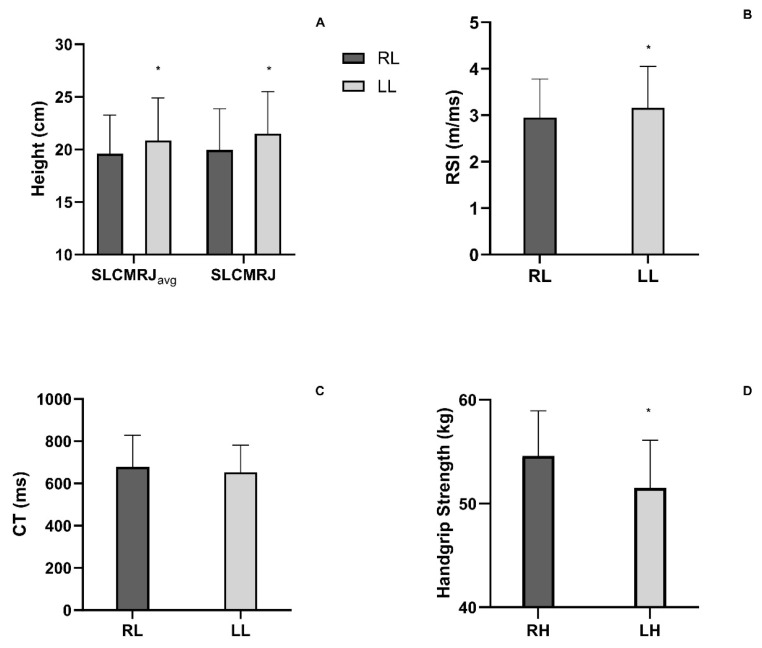
(**A**) Height performance of average and first Single-Leg Countermovement Rebound Jump (SLCMRJavg and SLCMRJ) of Right Leg (RL) and Left Leg (LL). (**B**) Reactive Strength Index (RSI) of Right Leg (RL) and Left Leg (LL). (**C**) Contact Time (CT) of Right Leg (RL) and Left Leg (LL). (**D**) Handgrip Strength of Right Hand (RH) and Left Hand (LH). * *p* < 0.001, statistical significance between right and left leg or hand.

Significant differences were found in the variables of the single-leg countermovement rebound jump (SLCMRJ) between the left and right legs. More specifically, the left leg had an 8.9 ± 15.9% higher SLCMRJ Avg (t = 3.494, *p* = 0.001), 12.0 ± 17.5% higher SLCMRJ (t = 4.259, d = 0.691, *p* = 0.001) and 7.8 ± 15.1% greater RSI (t = 2.93, *p* = 0.006) compared to the right leg (Figure 1A,B). However, no significant difference was found for contact time (CT) between legs (mean difference of −0.7 ± 13.4%; t = 0.966, d = 0.157, *p* = 0.340; Figure 1C). In addition, maximum HG strength was significantly higher for the right hand compared to the left hand (right: 54.4 ± 4.4 kg; left: 51.2 ± 4.9 kg; t = 6.123, *p* = 0.001; Figure 1D).

All external load variables showed statistically significant differences between seasonal phases, with the highest values generally observed during the pre-season or early in-season and the lowest during the offseason period (Table 2).

**Figure 2:**
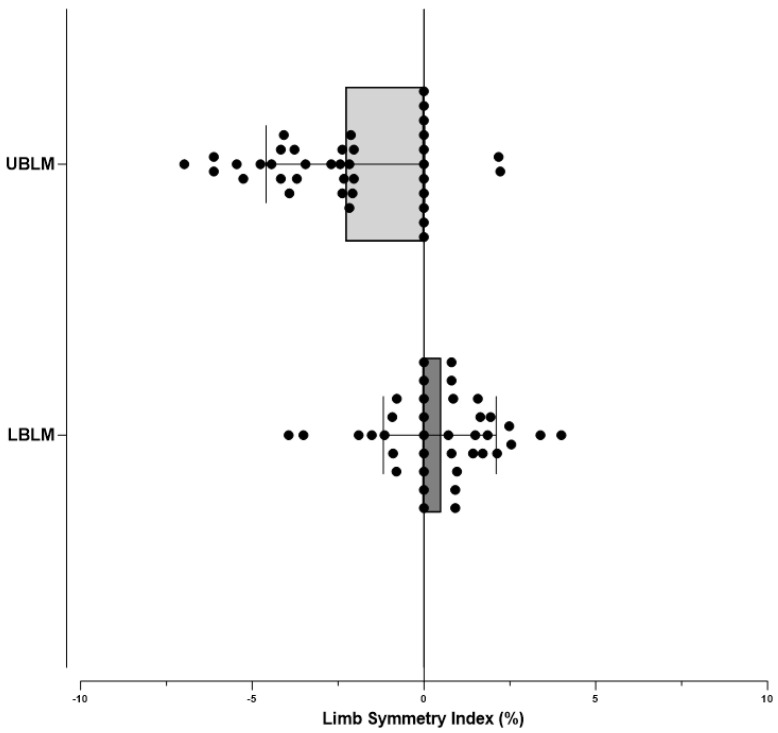
Limb Symmetry Index of Upper Body Lean Mass (UBLM) and Lower Body Lean Mass (LBLM).

Figure 2 illustrates the LSI for the UBLM and LBLM. The distribution of UBLM values showed greater variability, with the median shifted slightly toward negative values, indicating reduced symmetry compared to LBLM. In contrast, LBLM values were more tightly clustered around zero, with a narrower interquartile range, suggesting higher symmetry between limbs.

**Figure 3:**
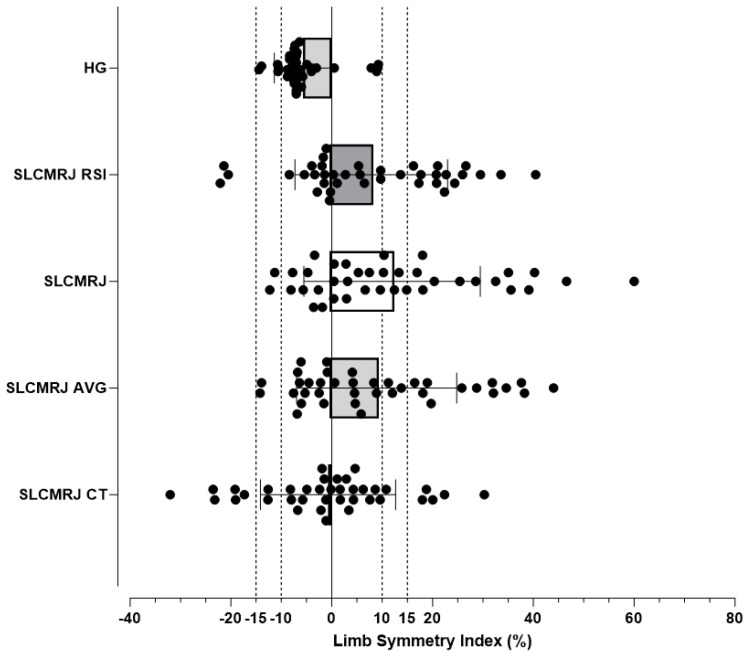
Limb Symmetry Index of Handgrip Strength (HG), Single-Leg Countermovement Rebound Jump (SLCMRJ), SLCMRJ Reactive Strength Index (RSI), SLCMRJ Average (AV), SLCMRJ Contact Time (CT).

Figure 3 presents the limb symmetry index (LSI) across different performance measures, including handgrip (HG) and single-leg countermovement jump (SLCMRJ) metrics. HG demonstrated the smallest variability, with values tightly clustered around zero, indicating minimal asymmetry. In contrast, SLCMRJ-related measures showed broader distributions, with several individuals exceeding the ±10% asymmetry threshold. The greatest variability was observed in SLCMRJ RSI and contact time, where numerous outliers extended beyond ±20%. These findings suggest that upper-body strength was relatively symmetrical, whereas lower-limb explosive and reactive measures revealed more pronounced asymmetries.

## 4. Discussion

The purpose of this study was to investigate structural and functional asymmetries between the upper and lower limbs of basketball players. Specifically, it was hypothesized that, in right-handed players, the left leg would demonstrate more favorable values in body composition characteristics, bone health parameters, and performance measures compared to the right leg. In contrast, the right arm was hypothesized to display more favorable structural and functional characteristics than the left. The present findings partially supported the hypothesis, revealing significantly higher lean mass, BMD, and BMC in the right arm compared to the left. However, no significant differences in lean mass of the lower limbs were observed, even though bone health parameters of the leg and femur were higher on the left side. In terms of performance, the left leg exhibited significantly greater SLCMRJ and RSI compared to the right leg, while the right arm showed greater HG strength than the left arm.

The findings of this study suggest that long-term participation in basketball may be associated with the development of functional and structural asymmetries, likely due to the sport’s repetitive, unilateral movements, such as lay-up shots, curvilinear sprinting, CODs, and jumping. However, research on this topic remains inconclusive. Ibáñez and his colleagues [24] reported no significant asymmetries in various game tasks, situations, or player positions when examining 12 professional male players from Spain’s top division. Their study, conducted during pre-season training sessions and unofficial matches, may have limited the detection of asymmetries due to the controlled environment and tactical adjustments reducing unilateral movement patterns. Therefore, it appears that their conclusions may not fully capture the asymmetries present in basketball players at different competitive levels or within diverse player populations [41]. Based on the above, additional research examined asymmetries in specific motor tasks related to basketball. For example, Gómez-Carmona et al. [23] identified significant asymmetries during curvilinear movements, where the outer leg experienced a greater load. This suggests that asymmetries may emerge in a task-specific manner based on the type of movement performed. In our sign convention, negative asymmetry values indicate cases where the non-dominant limb outperformed the dominant side; such directionality can reflect functional adaptations to habitual, task-specific loading (e.g., greater outside-leg demands during curved running and changes of direction) and should be interpreted with the task and metric in mind [24]. Similarly, Saucier [22] underlined the influence of positional demands on asymmetries through EMG analysis. Centers and forwards exhibited significant asymmetries during practice (46.0% L/54.0% R for centers; 46.4% L/53.6% R for forwards), which intensified further during games (44.2% L/55.8% R for forwards) meaning that exercise-induced fatigue may serve as an important factor exacerbating or even revealing inter-limb asymmetries, potentially increasing injury risk [42,43]. Fatigue impairs kinetic and kinematic symmetry by reducing vertical stiffness and loading rates during movement while increasing internal rotation and stiffness of the knee [42]. It has also been shown to increase asymmetries in SLCMJ markers after repeated sprints [9] or a soccer match, with recovery occurring within 48 and 72 h, respectively [42]. These findings highlight how specific basketball movements, positional differences, and exercise-induced fatigue can influence the development of asymmetry through repetitive, sport-specific motor patterns. However, post-match recovery kinetics vary between team sports. While recovery after a soccer match may exceed 72 h [44], in basketball, performance tends to return to normal within 48 h [39]. In addition, during congested schedules, this recovery time may be insufficient, as performance markers remain impaired for over 72 h after three consecutive basketball games [39]. This limited recovery capacity may contribute to the development or maintenance of asymmetries, although this remains unexplored in the basketball literature.

Among the numerous physical attributes linked to basketball performance, vertical jump height has proven to be a significant indicator. Evidence indicates its effectiveness in differentiating players based on competitive levels and positions. For example, Cui et al. found that jump height significantly distinguishes drafted NBA athletes from their non-drafted counterparts, underscoring its potential importance in talent identification [45]. Guards tend to exhibit greater bilateral symmetry in unilateral jumps compared to forwards and centers, with higher-level players generally showing more symmetrical performance than their lower-level counterparts [17]. Asymmetry in single-leg jumping has also been linked to increased injury risk, as demonstrated in youth soccer athletes [46]. Although landing forces were not directly assessed in that study, reduced single-leg jump performance may nonetheless indicate compromised neuromuscular function. In basketball, where repetitive high-intensity jumping and unilateral landings are frequent, asymmetrical players may be subjected to uneven mechanical loading, potentially elevating their injury risk [23]. The SLCMRJ, which incorporates landing metrics such as contact time and the reactive strength index (RSI), offers a practical means of detecting such asymmetries. Therefore, including single-leg power assessments in routine training and evaluation protocols may not only enhance performance monitoring but also contribute to targeted injury prevention strategies.

Although thresholds provide a useful reference for identifying potentially meaningful asymmetries, they should not be regarded as universal cut-offs. Instead, interpretation must remain individualized, as asymmetries may manifest differently across performance metrics (e.g., reactive strength index, jump height, lean mass). The task-specific nature of asymmetries suggests that a single threshold value may not adequately capture performance- or context-dependent differences. Future research using analytical approaches such as ROC curve analysis or subgroup comparisons may help establish more precise, task-specific thresholds.

For this reason, the present study examined lower limb power asymmetries, revealing significant differences in SLCMRJ performance. The left leg exhibited superior performance, with an 8.9 ± 15.9% higher mean SLCMRJ height compared to the right leg (*p* = 0.001). In addition, the left leg exceeded the right leg in maximum SLCMRJ height by 12.0 ± 17.5% (*p* = 0.001) and in RSI by 7.8 ± 15.1% (*p* = 0.006). Interestingly, no significant differences in (CT) were found between the legs. These findings align with prior research on the limb symmetry index (LSI). Schiltz et al. [21] reported an LSI of 12% in the drop jump (DJ) among professional basketball players, slightly higher than the 8.9% observed in our study. The findings of the present study align with the widely accepted 10% threshold associated with increased injury risk, consistent with reported values from basketball [18,26] and soccer [33], where single-leg countermovement jumps revealed asymmetry values of 10.4%, 11.3%, and 12.5%, respectively.

A critical consideration in the results was the inter-individual variability in asymmetry scores for jump metrics. For example, while the mean SLCMRJ asymmetry was 8.92%, the range extended from −14.20% to 44.00%. Similarly, RSI had an average asymmetry of 7.84%, ranging from −22.12% to 40.48% (Figure 3). These findings highlight the importance of conducting multidimensional assessments when detecting asymmetries, suggesting that detection thresholds may need to be adjusted based on the specific metrics assessed, such as SLCMRJ performance, compared to more traditional strength assessments.

While between-limb differences in SLCMRJ height and RSI indicate functional asymmetry, structural indices from BIA and DXA offer a complementary view: lower-limb lean mass did not differ significantly between sides, whereas DXA-derived bone parameters showed left-dominant asymmetry in the leg and hip (greater total-leg BMC: LSI = 1.7%, *p* = 0.002; higher trochanteric BMD: LSI = 1.8%, *p* = 0.031; higher total-hip BMD: LSI = 1.4%, *p* = 0.033). Clean mass was similar between sides (LSI: 0.46%; *p* = 0.080), and no significant difference was found in fat mass (LSI: −0.75% *p* = 0.584). It is worth noting that BMD values on both sides were above established population reference values for the same age group [47,48], indicating that the sample as a whole presented increased bone health, potentially as a result of chronic exposure to high-intensity activities such as basketball. Furthermore, during the four representative weeks of the season monitored in the present study, participants consistently accumulated a high number of jumps and multidirectional accelerations (Table 2). These movement patterns, observed in all phases of the season, reinforce the osteogenic nature of basketball, as they frequently provide mechanical loading stimuli promoting bone adaptation [49]. This general adaptation may also explain the observed asymmetries, which could reflect long-term osteogenic responses to unilateral loading. Supporting this, earlier research has shown that more experienced soccer players tend to have a greater tibial cross-sectional area and bone mass in the supporting leg due to repetitive high-impact and gravitational loads [50]. This pattern is observed in high-impact sports, where higher bone mineral density (BMD) is often found in the non-dominant or supporting limb. In contrast, in low-impact sports, such as swimming, BMD tends to be higher on the dominant side [7,49]. This phenomenon may also be influenced by the preferred limb use throughout the lifespan. Supporting this, Chilibeck et al. [51] reported that older individuals exhibit greater asymmetry between limbs compared to younger individuals, implicating that the cumulative effect of unilateral loading over time enhances structural imbalances. Regarding the structure of the hip region, previous studies in low-impact sports have reported that the dominant limb tends to display greater values at most proximal femoral regions, including the femoral neck, Ward’s triangle, and the shaft, excluding the trochanter. However, the present study suggests a different pattern, with the trochanter being the only region significantly larger in the left limb. This may be attributed to the demands of basketball-specific movements, which often involve lateral steps, unilateral loading during changes of direction, and rotational movements. These movements probably impose repetitive mechanical loading on the lateral hip structures, particularly the greater trochanter and associated muscles. Grimaldi and Fearon [52] suggested that these movements, especially when performed on a single-leg stance, increase shear forces in the trochanteric region due to tension in the gluteal tendons and iliotibial band. The absence of significant asymmetry in total lean leg mass observed in the present study may therefore suggest that muscular adaptations are more transient, responding predominantly to recent training stimuli rather than long-term cumulative loading across the lifespan [53].

Significant differences in upper limb strength were also observed, with the right hand displaying greater HG strength compared to the left (*p* = 0.001), corresponding to an LSI of 6.39%. However, this value is lower than the 13.6% asymmetry observed in elite female tennis players, which is likely due to the sport-specific demands of tennis, where force production with the dominant hand plays a key role, in contrast to basketball, which emphasizes shooting technique and ball handling skills [54]. Furthermore, asymmetric upper limb function has been associated with long-term adaptations to repetitive, dominant hand use in sports, particularly among athletes in overhead sports, such as baseball pitchers and tennis players [55,56]. The relevance of functional testing in detecting upper limb asymmetries and their impact on injury risk and in-season performance has been highlighted in studies involving athletes from multiple sports [57]. The risk of upper limb injury in basketball may occur not only from on-court activities, such as battling for rebounds, taking position, going for a steal, or a block, but also from off-court training activities, including resistance training.

Beyond functional capacity, structural asymmetries in the upper limbs were also pronounced. The right arm exhibited significantly greater lean mass than the left, with a 2.5% higher value (*p* = 0.001), accompanied by a 1.3% higher BMD (*p* = 0.010) and a 2.2% higher BMC (*p* = 0.012). These results are in agreement with McClanahan et al. [7], who reported significantly higher BMD in the dominant right arm across all male collegiate sports teams, with side-to-side differences ranging from 4.4% in basketball to 17.6% in tennis. The predominance of the right arm in mechanical loading, particularly in sports such as basketball and volleyball, appears sufficient to elicit such osteogenic adaptations, as reflected in increased BMD values.

To further investigate the magnitude and direction of these asymmetries, a segmental phase angle analysis was conducted, revealing significant asymmetries in both the arms and legs. Specifically, the right arm showed a 4.3% higher phase angle compared to the left (Arms: Left 6.82° ± 0.67°, Right 7.11° ± 0.62°, *p* = 0.001), while the right leg exhibited a 1.4% higher phase angle relative to the left (Legs: Left 6.28° ± 0.67°, Right 6.37° ± 0.68°, *p* = 0.040). In the upper limbs, phase angle asymmetry clearly mirrored the pattern observed in lean mass, bone health, and functional performance, with consistently greater values on the right side. These findings strengthen the validity of the phase angle as a physiological indicator of segmental asymmetry, reflecting structural and functional laterality, which aligns with the findings of Carrasco-Fernández et al. [58], who also found similar phase angle imbalances in elite female handball players. Nevertheless, the findings in the lower limbs were less consistent. Although a statistically significant difference was found, the magnitude of asymmetry was lower and had the opposite direction to performance and bone health metrics. This discrepancy implies that the phase angle may reflect different aspects of tissue quality or may align with the broader concept of short-term and long-term physiological adaptations.

The observed asymmetries suggest a possible relationship with performance metrics. Interestingly, no significant correlations were found between lower limb lean mass asymmetries and performance outcomes from the SLCMRJ, including average jump height (r = 0.003, *p* = 0.984), jump height (r = −0.05, *p* = 0.767), contact time (r = 0.094, *p* = 0.573), and RSI (r = −0.037, *p* = 0.824). This contrasts with the research of Bell et al. [12], who suggested that lower body lean mass asymmetries could account for some variance in force and power during jumping, with the LSI of lean mass in key areas explaining 20–25% of the variance. However, a significant portion of this variance remains unexplained, implying the complexity of performance outcomes. Lower lean body mass has been linked to factors such as energy absorption during landing, knee laxity, and reduced stiffness, particularly in females [21,59]. Although lean mass contributes to strength and power asymmetries, these findings underscore the multifactorial nature of performance, suggesting that variables like strength-to-body mass ratio and neuromuscular control are critical in influencing both performance and injury risk during vigorous activities [59,60]. While the data reveals structural asymmetries, the relationship between these asymmetries and performance remains complex, indicating that additional factors must be considered for a comprehensive understanding of athletic performance.

Neural factors, including variations in motor unit recruitment patterns, firing rates, and neuromuscular coordination, may play a significant role in performance asymmetries [61]. These neural components can affect how structural imbalances manifest functionally, potentially influencing the relationships between muscle mass, strength, and power. Given the sport-specific movement patterns in basketball, such as unilateral jumps, directional changes, and repetitive shooting, these asymmetries could be reinforced or even developed over time [9]. However, this study did not delve into neurophysiological mechanisms, and these considerations remain speculative. Additionally, we did not prospectively track time-loss or medical-attention injuries; therefore, despite observing inter-limb asymmetries, we cannot infer their relationship to injury occurrence or risk.

Certain limitations to this study should be acknowledged. First, no direct power output measurements were performed, as a contact mat was used instead of a force plate, which may have reduced the precision of power-related assessments. Additionally, the testing battery did not include other relevant performance measures such as agility, reactive agility, or balance. While handgrip provides reliable estimates of isometric capacity and local side-to-side asymmetry, it is not a surrogate for basketball-specific upper-limb asymmetry, which is multidimensional, direction-dependent, and distributed across the kinetic chain; therefore, handgrip asymmetry should not be generalized to global or task-level asymmetry in basketball. Future studies should aim to address these limitations by incorporating more comprehensive testing protocols and considering positional groupings to elucidate better the nature and implications of asymmetries in basketball athletes.

A further limitation is that correlation analyses were restricted to Pearson’s r, which assumes linear associations and does not account for potential threshold effects or nonlinear dynamics. Additionally, covariates that may influence the relationship between structural and functional asymmetries (e.g., training exposure, injury history, or positional demands) were not included. Future studies employing regression models, nonlinear approaches, or unsupervised methods (e.g., clustering) may provide deeper insights into the complexity of these relationships.

## 5. Conclusions

This study examined both structural and functional asymmetries in basketball players, revealing upper-limb differences that favored the dominant arm and lower-limb differences that favored the non-dominant leg. These patterns align with the specific loading demands of the sport, but within this cross-sectional framework, we cannot confirm them as being training-induced. The measurements related to bone displayed consistent directional asymmetries, indicating potential long-term adaptations, while asymmetries in lean tissue and performance were less uniform and may reflect more transient influences. The detection of these asymmetries through power-based tests underscores their utility in identifying performance-relevant differences, although their effectiveness compared to other methods has not been assessed. Overall, these findings underscore the importance of context-specific interpretations of asymmetries and advocate for the use of complementary structural and functional assessments to enhance athlete monitoring and inform individualized training decisions.

## Figures and Tables

**Table 1:** Descriptive Statistics of Anthropometric and Performance Variables Assessed via Charder MA801 Bioelectrical Impedance Analyzer.

	Mean	Standard Deviation	Range (Min/Max)
Weight (kg)	86.18	9.09	64.39–98.20
Height (cm)	191.59	8.15	175.00–212.00
Age (years)	20.94	2.71	15.55–28.92
Body Mass Index (BMI, kg/m^2^)	23.50	2.26	18.80–29.70
Fat Mass (FM, kg)	11.20	4.61	4.60–22.10
Fat-Free Mass (FFM, kg)	75.00	7.12	59.20–89.70
Percentage Body Fat (PBF, %)	12.77	4.49	6.10–22.90
Countermovement Rebound Jump Average (CRMJ Avg, cm)	41.20	6.43	29.30–58.70
Contact Time Average (CT Avg, ms)	597.40	91.64	452.00–787.00

**Table 2:** Seasonal Variations in External Load Metrics Across the Annual Training Cycle.

Metric	Pre-Season	Early In-Season	Late In-Season	Transition	F (_3, 51_)	*p* Value	η^2^
AAL (AU)	4142.4 ± 566.9	3599.6 ± 507.5 ^a^	3312.9 ± 403 ^a, b^	2525.4 ± 395.1 ^a,b,c^	75.19	0.000	0.816
Distance (m)	29,038.9 ± 3848 ^a^	23,937.7 ± 2728.2 ^a^	23,037 ± 2486 ^a^	3474.6 ± 400.88 ^a,b,c^	473.94	0.000	0.965
Mechanical Load (AU)	10,402 ± 860 ^a^	9189.5 ± 1064 ^a^	8481.4 ± 893.1 ^a^	6506.4 ± 1015.5 ^a,b,c^	89.94	0.000	0.841
Jump Load (J)	116,961 ± 41,203 ^a^	149,657 ± 48,297 ^b^	137,410 ± 47,999 ^b^	22,061 ± 7179 ^a,b,c^	58.1	0.000	0.774
Jumps (Events)	479.3 ± 143.1 ^b^	600.9 ± 188.1	547.9 ± 180.6	451.8 ± 137.77 ^b,c^	8.16	0.000	0.324

Data are presented as means ± SD. a: Different from Pre-season, b: Different from Early In-Season, c: Different from Late In-Season.

**Table 3:** Comparison of Segmental Body Composition and Phase Angle Between Left and Right Limbs Assessed via Charder MA801 Bioelectrical Impedance Analyzer.

	Left Side	Right Side	t Value	*p* Value	Cohen’s d
50 kHz Phase Angle Arms (°)	6.82 ± 0.67	7.11 ± 0.62	6.130	0.000	0.17
50 kHz Phase Angle Legs (°)	6.28 ± 0.67	6.37 ± 0.68	2.123	0.040	0.21
Lean Mass Arms (kg)	4.43 ± 0.53	4.54 ± 0.57	6.047	0.000	0.11
Lean Mass Legs (kg)	11.85 ± 1.30	11.79 ± 1.25	−1.801	0.080	0.21
Fat Mass Arms (kg)	0.42 ± 0.24	0.43 ± 0.24	0.813	0.422	0.04
Fat Mass Legs (kg)	1.45 ± 0.77	1.44 ± 0.75	−0.552	0.584	0.09

**Table 4:** Comparison of Bone Mineral Density (BMD) and Bone Mineral Content (BMC) Between Left and Right Limbs and Hip Regions Assessed via Dual Energy X-ray Absorptiometry.

Variable	Left Side	Right Side	t Value	*p* Value	Cohen’s d
BMD Total Arms (g/cm^2^)	1.015 ± 0.076	1.028 ± 0.081	−2.733	0.010	0.03
BMD Total Legs (g/cm^2^)	1.621 ± 0.107	1.605 ± 0.109	1.782	0.083	0.06
BMC Total Arms (g)	265.187 ± 40.288	271.132 ± 41.485	−2.635	0.012	13.86
BMC Total Legs (g)	816.233 ± 105.631	801.421 ± 106.876	3.339	0.002	26.35
BMD Neck (g/cm^2^)	1.418 ± 0.156	1.426 ± 0.157	−0.743	0.462	0.07
BMD Upper Neck (g/cm^2^)	1.319 ± 0.185	1.333 ± 0.184	−1.009	0.319	0.08
BMD Lower Neck (g/cm^2^)	1.514 ± 0.150	1.518 ± 0.150	−0.269	0.790	0.08
BMD Ward’s (g/cm^2^)	1.327 ± 0.178	1.333 ± 0.203	−0.381	0.705	0.09
BMD Trochanter (g/cm^2^)	1.216 ± 0.137	1.192 ± 0.138	2.248	0.031	0.07
BMD Shaft (g/cm^2^)	1.621 ± 0.169	1.608 ± 0.163	1.216	0.232	0.07
BMD Total Hip (g/cm^2^)	1.424 ± 0.154	1.402 ± 0.146	2.216	0.033	0.06
BMC Neck (g)	8.146 ± 1.072	8.248 ± 1.160	−1.042	0.304	0.60
BMC Upper Neck (g)	3.731 ± 0.558	3.794 ± 0.617	−1.234	0.225	0.31
BMC Lower Neck (g)	4.415 ± 0.574	4.454 ± 0.590	−0.688	0.496	0.35
BMC Ward’s (g)	4.901 ± 1.041	4.984 ± 1.115	−0.693	0.493	0.73
BMC Trochanter (g)	20.265 ± 3.156	19.947 ± 3.172	1.098	0.279	1.77
BMC Shaft (g)	26.520 ± 2.928	26.351 ± 2.987	0.821	0.417	1.27
BMC Total Hip (g)	54.880 ± 5.986	54.545 ± 6.305	0.784	0.438	2.63

## Data Availability

The data from this article will be made available by the authors on reasonable request.
